# Tissue-resident Lymphocytes Are Released During Hypothermic and Normothermic Machine Perfusion of Human Donor Kidneys

**DOI:** 10.1097/TP.0000000000004936

**Published:** 2024-04-01

**Authors:** Daphne M. Hullegie-Peelen, Dennis A. Hesselink, Marjolein Dieterich, Robert C. Minnee, Annemiek Peeters, Martin J. Hoogduijn, Carla C. Baan

**Affiliations:** 1 Department of Internal Medicine, Nephrology and Transplantation, Erasmus Medical Center Transplant Institute, University Medical Center Rotterdam, Rotterdam, the Netherlands.; 2 Division of Hepato-pancreatobiliary and Transplant Surgery, Department of Surgery, Erasmus MC Transplant Institute, University Medical Center Rotterdam, Rotterdam, the Netherlands.

## Abstract

**Background.:**

Machine perfusion is the preferred preservation method for deceased donor kidneys. Perfusate fluid, which contains a complex mixture of components, offers potential insight into the organ’s viability and function. This study explored immune cell release, particularly tissue-resident lymphocytes (TRLs), during donor kidney machine perfusion and its correlation with injury markers.

**Methods.:**

Perfusate samples from hypothermic machine perfusion (HMP; n = 26) and normothermic machine perfusion (NMP; n = 16) of human donor kidneys were analyzed for TRLs using flow cytometry. Residency was defined by expressions of CD69, CD103, and CD49as. TRL release was quantified exclusively in NMP. Additionally, levels of cell-free DNA, neutrophil gelatinase-associated lipocalin, and soluble E-cadherin (sE-cadherin) were measured in NMP supernatants with quantitative polymerase chain reaction and enzyme-linked immunosorbent assay.

**Results.:**

Both HMP and NMP samples contained a heterogeneous population of TRLs, including CD4^+^ tissue-resident memory T cells, CD8^+^ tissue-resident memory T cells, tissue-resident natural killer cells, tissue-resident natural killer T cells, and helper-like innate lymphoid cells. Median TRL proportions among total CD45^+^ lymphocytes were 0.89% (NMP) and 0.84% (HMP). TRL quantities in NMP did not correlate with donor characteristics, perfusion parameters, posttransplant outcomes, or cell-free DNA and neutrophil gelatinase-associated lipocalin concentrations. However, CD103^+^ TRL release positively correlated with the release of sE-cadherin, the ligand for the CD103 integrin.

**Conclusions.:**

Human donor kidneys release TRLs during both HMP and NMP. The release of CD103^+^ TRLs was associated with the loss of their ligand sE-cadherin but not with general transplant injury biomarkers.

## Graphical abstract

**Figure:**
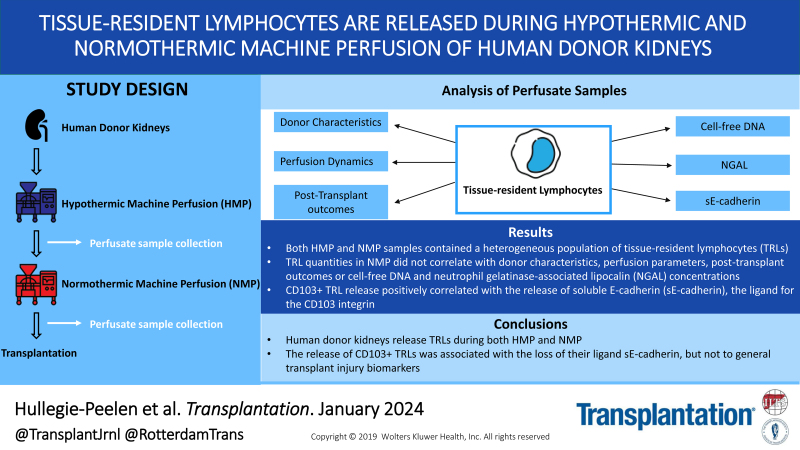

## INTRODUCTION

Machine perfusion has become the preferred method for preserving deceased donor organs.^1^ Currently, 2 prominent types exist: hypothermic machine perfusion (HMP), which maintains a low metabolic state, and normothermic machine perfusion (NMP), which activates metabolic processes and allows for functional assessment.^1,2^ Perfusates collected during HMP and NMP contain soluble factors, extracellular vesicles, and donor-derived cells and may inform on the organ’s viability and function before transplantation.^3–5^ However, none of these components has shown sufficient prognostic utility.^5,6^ Various immune cells originating from the organ are also released during machine perfusion, but their relation with organ’s viability and graft outcomes is currently also unclear.^5,7-9^ Notably, even tissue-resident lymphocytes (TRLs) have been identified in perfusates of donor organs despite their usual residency within tissues.^10-12^

TRLs constitute a diverse group of cells residing in nonlymphoid tissues like kidneys and play a key role in local immune surveillance.^13,14^ TRLs are characterized by the expression of specific residency markers, such as CD69, CD103, and CD49a.^14,15^ Their release in perfusates is intriguing concerning their usual residency within tissue and might be a response to cellular stress or tissue injury. Consequently, exploring TRL prevalence in donor kidney perfusates could provide a noninvasive approach to assess organ viability pretransplantation.

This study aimed to investigate the presence, quantity, and characteristics of TRLs in donor kidney perfusates during HMP and NMP. Furthermore, their correlation with tissue injury markers, pretransplant donor characteristics, perfusion dynamics, and posttransplant graft outcomes was investigated.

## MATERIALS AND METHODS

### Study Design and Sample Collection

**FIGURE 1.**
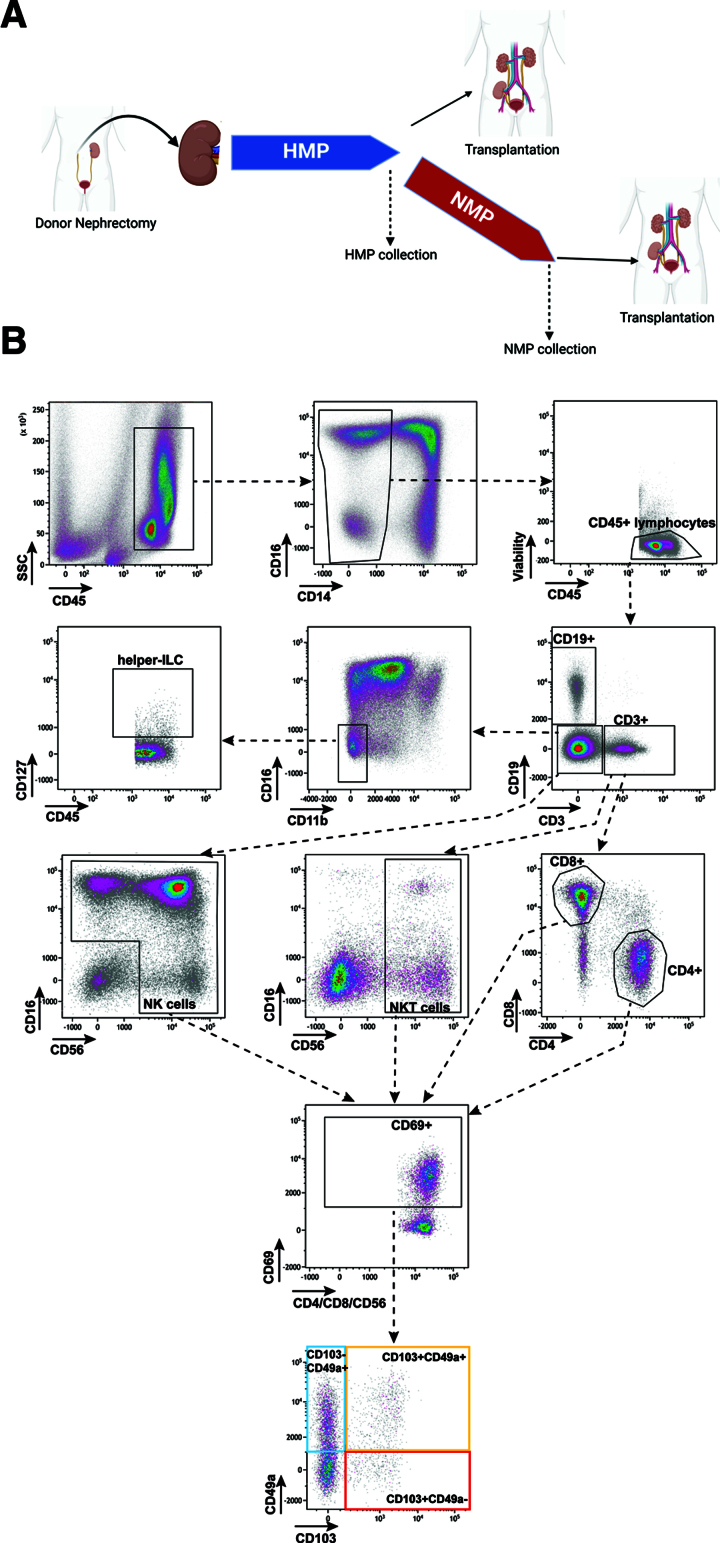
Study overview and gating strategy. Donor kidneys underwent HMP with or without additional NMP; samples were collected after both procedures. Created with BioRender.com (A). Gating strategy for flow cytometric analysis of immune cells, including tissue-resident lymphocytes in HMP and NMP samples. Within the CD45^+^ lymphocyte population, general immune cell subsets were delineated as CD3^+^ (encompassing both CD4^+^ and CD8^+^ T cells), CD19^+^ B cells, NK cells, and NKT cells. Further analysis within the subsets of CD3^+^CD4^+^ T cells, CD3^+^CD8^+^ T cells, NK cells, and NKT cells focused on assessing the expression of tissue residency markers: CD69, CD103, and CD49a. The populations framed in blue, yellow, and red are identified as tissue-resident lymphocytes. Additionally, the resident helper-like ILC population was characterized as viable CD45^+^ lineage-(CD3^−^CD19^−^CD11b-CD16^−^)CD127^+^ cells (B). HMP, hypothermic machine perfusion; ILC, innate lymphoid cell; NK, natural killer; NKT cell, NK T cell; NMP, normothermic machine perfusion.

This ancillary study involved the prospective collection of kidney perfusates obtained during HMP and NMP of donor kidneys enrolled in the APOLLO clinical trial (NCT04882254).^16^ The sample collection took place at the Erasmus MC Transplant Institute, University Medical Center, Rotterdam, the Netherlands, from April 2021 to July 2023. Donor kidneys that were from donation after circulatory death (DCD) donors or donation after brain death (DBD) donors fulfilling the expanded criteria donor criteria were included in the APOLLO trial. All donor kidneys underwent HMP using a University of Wisconsin preservation solution. Additionally, kidneys in the trial’s intervention arm underwent a supplementary 120-min NMP cycle (**Figure S1, SDC**, http://links.lww.com/TP/C970).^16^ The NMP perfusate primarily consisted of leukocyte-depleted packed red blood cells (RBCs), matched to the recipient’s blood type.^16^ HMP samples originated from either the trial’s control arm or from routine kidney transplantations. All samples were collected at the end of perfusion (Figure 1A). Detailed information regarding the HMP and NMP procedures can be found elsewhere.^16^ Posttransplant outcome measures included delayed graft function (DGF), primary nonfunction (PNF), and acute rejection (AR). DGF was defined as the need for dialysis within the first week after transplantation. AR was defined as a biopsy-proven AR according to the Banff 2019 classification.^17^

### Study Approval

Ethical approval for the APOLLO clinical trial and the use of biobank materials was obtained from the Medical Ethical Review Board of the Erasmus MC, Rotterdam, the Netherlands (MEC-2020-0366 and MEC-2010-022). All experimental procedures aligned with the guidelines and regulations set by our institution. All patients gave written informed consent.

### Cell Isolation From HMP and NMP Samples

On collection, the perfusates were processed within 48 h. For cellular analysis of HMP and NMP samples, 100 mL of perfusate was used. To concentrate the leukocytes, either a centrifugation step or a standard Ficoll isolation procedure was performed.^18^ Subsequently, the cell suspensions were analyzed using flow cytometry following the protocol described below.

To acquire supernatants from both HMP and NMP samples, centrifugation was carried out at 1972*g* for 10 min. The resultant supernatant was then aliquoted into vials and stored at –80 °C for future analysis.

For comparative purposes, peripheral blood mononuclear cells (PBMCs) from healthy individuals and packed RBCs were included. PBMCs were obtained from the biobank and the packed RBCs were obtained from the blood transfusion laboratory, both part of the Erasmus MC, Rotterdam, the Netherlands. The RBCs were deemed unsuitable for transfusion because of extended time outside the transfusion laboratory and were then allocated for research purposes.

### Flow Cytometry Analysis

Fresh cell suspensions were stained with fixable-viability 780 staining (BD Biosciences, Franklin Lakes, NJ) for 15 min at room temperature in the dark, followed by 1 centrifugation cycle at 877*g* for 5 min. This was followed by a 30-min incubation with fluorescent antibodies targeting TRL markers (**Table S1, SDC**, http://links.lww.com/TP/C970). Residency was assessed by the coexpression of CD69 with CD103 or CD49a. No additional residency markers were used for helper-like innate lymphoid cells (ILCs). The gating strategy for cell subsets is presented in Figure 1B.

All samples were measured using a FACSymphony A3 Cell Analyzer (BD Biosciences), and the resultant data were analyzed using Kaluza Analysis version 2.1 software (Beckman Coulter, Brea, CA). Quantitative analysis, calculating absolute TRL numbers per 100 mL (from a total volume of 1 L), was conducted exclusively for NMP samples. HMP sample quantification was not feasible because of unrecorded total perfusion volumes.

### Cell-free DNA Assay

Cell-free DNA (cfDNA) was isolated from a minimum of 2 mL of NMP supernatant using the Circulating Nucleic Acid Kit from QIAGEN (Hilden, Germany) following the manufacturer’s instructions. Glyceraldehyde-3-phosphate dehydrogenase (GAPDH) gene expression, representing nuclear DNA, was quantified using the GAPDH Taqman gene expression assay (assay ID: Hs03929097_g1) from Thermo Fisher Scientific (Waltham, MA). GAPDH levels were measured on the StepOnePlus Real-Time Quantitative Polymerase Chain Reaction (qPCR) System (Thermo Fisher Scientific). Gene expression was calculated by transforming the cycle threshold (Ct) to cDNA copies (2^^(45–Ct)^).

### Enzyme-linked Immunosorbent Assays

Concentrations of neutrophil gelatinase-associated lipocalin (NGAL) and soluble E-cadherin (sE-cadherin) in NMP supernatants were assessed using enzyme-linked immunosorbent assay kits from Thermo Fisher Scientific and R&D Systems (Minneapolis, MN), respectively, adhering to the manufacturers’ protocols. Packed RBCs were included as background references. All samples, controls, and standard dilutions were measured in duplicate. NGAL samples underwent a 1:50 dilution and sE-cadherin a 1:10 dilution. Absorbance was measured at 450 nm using a Wallac 1420 Victor2 microplate reader (Perkin Elmer, Waltham, MA).

### Statistical Analysis

For statistical evaluation, GraphPad Prism version 9 (GraphPad Software, La Jolla, CA) was used. Data distribution was assessed using the Kolmogorov-Smirnov test, revealing normal distribution of all data. Therefore, only nonparametric tests were applied. The Mann-Whitney *U* test was selected for 2-group comparisons, and the Kruskal-Wallis test with the Dunn post hoc test for >2 groups. Group proportions were compared using the Fisher exact test. Correlation was analyzed using the Spearman rank correlation (ρ). *P* values <0.05 were considered statistically significant. In flow cytometry analysis, subset proportions were only calculated if the primary population exceeded 50 cells.

## RESULTS

### A Diverse Population of TRLs Is Released During Machine Perfusion of Donor Kidneys

To investigate whether immune cell populations are released from donor kidneys during machine perfusion, flow cytometric analysis of 26 HMP and 16 NMP perfusates was performed (Figure 1; **Figure S1, SDC**, http://links.lww.com/TP/C970). Baseline characteristics were comparable between the HMP and NMP samples (**Table S2, SDC**, http://links.lww.com/TP/C970).

**FIGURE 2.**
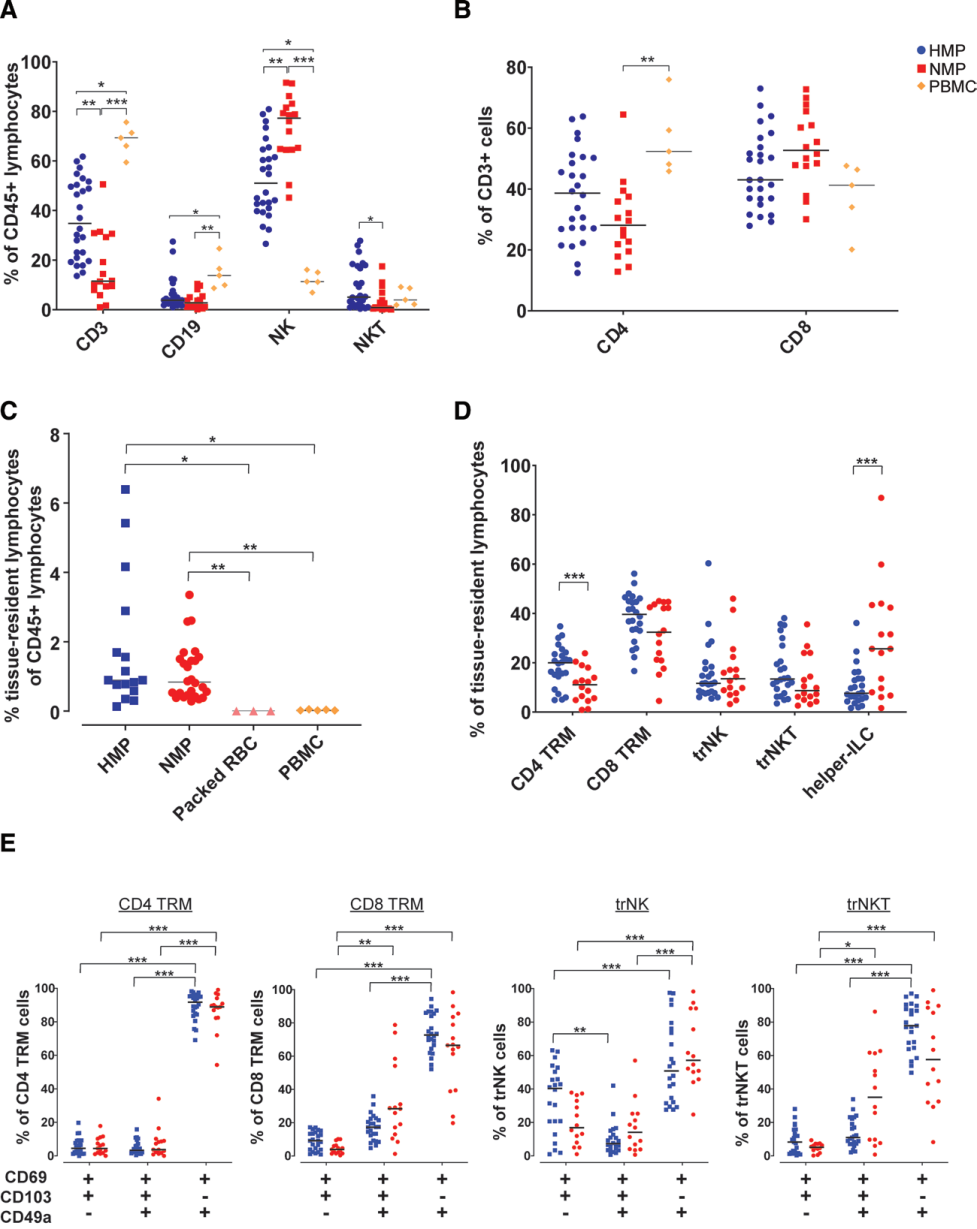
Prevalence of tissue-resident lymphocytes in perfusates. Analysis of CD3, CD19, NK, and NKT cell populations as a percentage of CD45^+^ lymphocytes in HMP (n = 26), NMP (n = 16), and PBMC (n = 5) samples (A). The proportions of CD4^+^ and CD8^+^ cells among the CD3^+^ cells across different sample types (B). Evaluation of TRLs as a proportion of CD45^+^ lymphocytes in HMP (n = 26), NMP (n = 16), packed RBC (n = 3), and PBMC (n = 5) samples (C). Proportion of different TRL subpopulations in HMP and NMP samples, represented as a percentage of total TRLs (D). The expression of tissue residency markers within these TRL subpopulations, presented as a percentage of the respective TRL subpopulation. E, Horizontal bars represent the median. The Kruskal-Wallis test with Dunn’s multiple comparison was used to examine statistical significance. *P < 0.05, **P < 0.01, and ***P < 0.001. HMP, hypothermic machine perfusion; NK, natural killer; NKT cell, NK T cell; NMP, normothermic machine perfusion; PBMC, peripheral blood mononuclear cell; RBC, red blood cell; TRL, tissue-resident lymphocyte.

Initial examination of HMP and NMP perfusates showed notable variations in CD3^+^, CD19^+^, natural killer (NK) cell, and NKT cell proportions compared with PBMCs from healthy subjects (Figure 2A). Both HMP and NMP contained fewer CD3^+^ and CD19^+^ cells, but a higher prevalence of NK cells, compared with PBMCs. Within the CD3^+^ population, NMP perfusates had lower CD4^+^ proportions compared with PBMCs (Figure 2B).

Further analyses focused on the proportion of TRLs within the living CD45^+^ lymphocyte population based on CD69, CD103, and CD49a expression (Figures 1B and 2C). The median TRL proportions in HMP and NMP samples were comparable (0.89% and 0.84%, respectively), with virtually no TRLs detected in packed RBC and PBMC controls (medians: 0.00% and 0.02%, respectively). Notably, NMP samples exhibited a significantly higher proportion of helper-ILCs than HMP samples, whereas HMP samples had a higher proportion of CD4^+^ tissue-resident memory T cell (T_RM_ cells) compared with NMP samples (Figure 2D). Proportions of other TRL subsets were comparable between both groups. Most TRLs displayed a CD69^+^CD49a^+^CD103^−^ phenotype, yet some exhibited CD103 expression, with or without CD49a (Figure 2E).

### No Association Between TRL Release and Donor Characteristics, Perfusion Parameters, or Posttransplant Graft Outcomes

Quantitative analysis was exclusively feasible for NMP perfusates because ofhe unrecorded total perfusion volume in HMP procedures. Absolute cell numbers of each TRL subpopulation in NMP perfusates are depicted in **Figure S2** (**SDC**, http://links.lww.com/TP/C970).

To determine underlying factors contributing to TRL release, comparisons were made between the absolute TRL quantity in NMP perfusates and various donor characteristics and perfusion parameters. Donor age, donor serum creatinine concentrations at explantation, donor BMI, kidney weight, cold ischemia time, type of donor (DBD/DCD), and a prior history of hypertension displayed no association with TRL cell numbers in NMP samples (**Figure S3, SDC**, http://links.lww.com/TP/C970). Additionally, renal end flow and intrarenal resistance displayed no correlation with TRL quantities in NMP samples (**Figure S3, SDC**, http://links.lww.com/TP/C970).

Next, potential associations between TRLs release and posttransplant outcomes were explored by assessing the incidence of DGF and AR and estimated glomerular filtration rate values posttransplantation. No difference in the TRL quantities in NMP perfusates was observed between kidneys with and without DGF (**Figure S4, SDC**, http://links.lww.com/TP/C970). A singular event of an AR within the initial posttransplant month and only 1 case of PNF occurred, leaving any potential correlation between PNF or AR and TRLs release inconclusive. No discernible association was found between estimated glomerular filtration rate levels posttransplantation and TRL release (**Figure S4, SDC**, http://links.lww.com/TP/C970).

### TRL Release in Donor Kidney Perfusates Is Correlated With sE-cadherin Release, But Not With Tissue Injury Biomarkers

**FIGURE 3.**
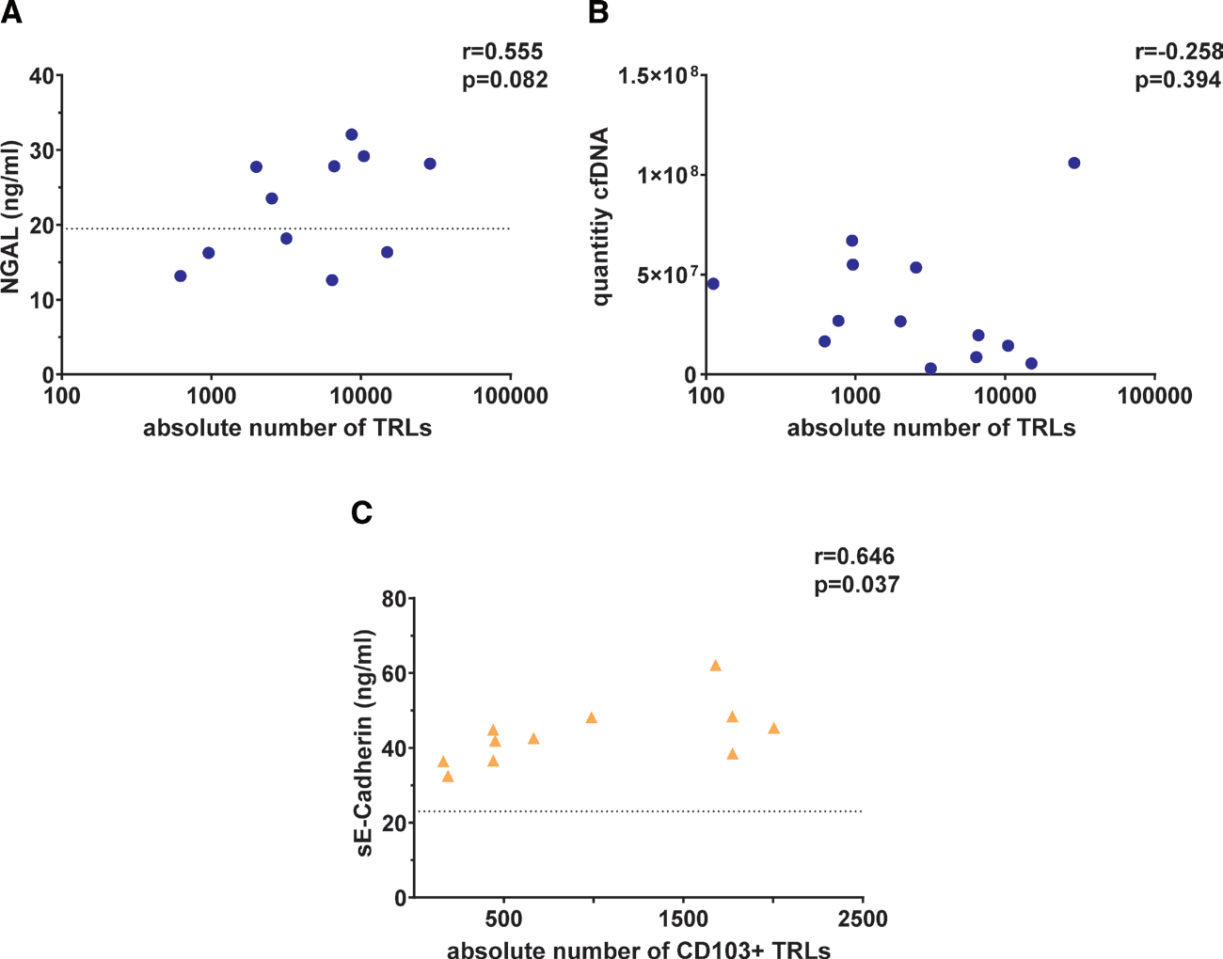
Potential biomarkers of tissue injury. The quantity of TRLs in NMP samples was not correlated with the levels of NGAL (A) or cfDNA (B). The quantity of CD103^+^ TRLs in NMP samples was positively correlated with the levels of sE-cadherin. The dotted lines represent the baseline reference of packed RBC (A and C); the reference is not visible in (B), due to the range of cfDNA levels and scale of the y-axis. Spearman’s correlation was used for correlation analysis (A–C). *r* = correlation coefficient. *P < 0.05, **P < 0.01, ***P < 0.001. cfDNA, cell-free DNA; NGAL, neutrophil gelatinase-associated lipocalin; NMP, normothermic machine perfusion; RBC, red blood cell; sE-cadherin, soluble E-cadherin; TRL, tissue-resident lymphocyte.

The association between TRL release in NMP perfusates and potential tissue injury biomarkers was subsequently investigated. Therefore, cfDNA and NGAL concentrations were measured in available perfusate supernatants (n = 13 and n = 11, respectively; **Figure S1, SDC**, http://links.lww.com/TP/C970). For 3 and 5 donor kidneys, no NMP supernatant was available for cfDNA and NGAL experiments, respectively. Notably, NGAL levels were below the packed RBC reference baseline, with no observed correlation between NGAL levels and TRL quantities in the remaining 6 samples (Figure 3A). In contrast, cfDNA was detected in each NMP supernatant, but no correlation with TRL quantities was observed (Figure 3B). A notable exception was 1 sample showing markedly elevated levels of both cfDNA and TRLs (Figure 3B). Noteworthy, this sample was derived from the patient who manifested with both PNF and AR.

The potential loss of TRL retention ligands as an explanation for TRL release in NMP perfusates was examined by measuring sE-cadherin—the ligand for the CD103 retention molecule—in the available NMP supernatants (n = 11; **Figure S1, SDC**, http://links.lww.com/TP/C970). sE-cadherin was present in all NMP supernatants, and its concentrations were markedly above the packed RBC reference baseline. Intriguingly, although no correlation was identified between sE-cadherin concentrations and the overall quantity of TRLs (*r* = 0.40, *P* = 0.23, data not shown), a significant association existed between the sE-cadherin concentrations and the quantity of CD103^+^ TRLs released (*r* = 0.65, *P* = 0.037; Figure 3C). This suggests that the release of CD103^+^ TRLs in perfusates might be triggered by loss of their retention ligand from kidney tissue.

## DISCUSSION

Machine perfusion of deceased donor kidneys enables investigation of the components released by the donor organ in an isolated setting and the study of their potential relation with organ viability before transplantation.^3–6^ In this study, it is demonstrated that during both HMP and NMP, donor kidneys release immune cells, including a diverse population of TRLs. The number of released TRLs did not exhibit correlations with donor characteristics, perfusion dynamics, transplant outcomes, or the levels of the injury markers cfDNA and NGAL. However, a positive correlation was observed between the release of CD103^+^ TRLs and their associated retention molecule ligand, sE-cadherin.

The finding that TLRs are released in perfusates is noteworthy, given their typical long-term residency within tissues.^15^ In livers, TRLs residing in the sinusoids are believed to be easily flushed into perfusates.^19^ However, unlike livers, kidneys lack such sinusoids and harbor TRLs only in deeper tissue compartments.^15^ This anatomical difference theoretically suggests that TRLs remain within the kidney tissue during perfusion. Contrary to this expectation, our findings align with a study that noted the presence of tissue-resident (tr)NK cells in human kidney perfusates.^12^ In addition to trNK cells, we also observed CD4^+^ T_RM_, CD8^+^ T_RM_, trNKT cells, and helper-ILCs in the perfusate samples. The release of this diverse population of TRLs was unrelated to donor characteristics, perfusion parameters, and posttransplant outcomes. In contrast, a previous study reported a negative correlation among resident immune cell release and cold ischemia time. It was hypothesized that ischemic injury results in activation and infiltration of immune cells.^10^ Here, no such relation was observed. In this previous study, vascular resistance was also correlated with the release of typically fixed structural liver cells.^10^ No correlation between vascular resistance or other perfusion parameters and TRL release was observed in the present study.

The investigation into the relationship between TRL release and tissue injury biomarkers (cfDNA and NGAL) demonstrated no significant associations. Although the efficacy of NGAL as an injury marker during machine perfusion of organ transplants has shown inconsistent results, cfDNA has been more consistently reported as a feasible injury marker.^5,6,20-22^ The lack of correlation between cfDNA and TRL release thus suggests that TRL release is not associated with tissue injury. An intriguing case displayed elevated concentrations of both cfDNA and TRLs. This particular donor kidney developed PNF and AR posttransplantation. However, without additional PNF and AR cases, it is uncertain whether this observed phenomenon is coincidental or indicative of a biological correlation.

A noteworthy observation was the significant association between sE-cadherin concentrations and the release of CD103^+^ TRLs in perfusates, suggesting that changes in adhesion dynamics might play a pivotal role in TRL release during perfusion. Prior studies have indicated that ischemia–reperfusion injury may trigger E-cadherin release.^23,24^ Considering that all deceased donor kidneys experience ischemia and NMP may mimic reperfusion effects, it is plausible that the perfused kidneys experienced ischemia–reperfusion injury-like injury, leading to E-cadherin release.^25,26^ However, the positive correlation between E-cadherin and CD103^+^ TRLs release requires confirmation in a larger cohort of samples.

Another scenario is that external factors trigger TRLs to actively migrate from the tissue into the perfusate. Downregulation of CD69 is required for active tissue egress.^27^ The TRLs identified in perfusates expressed CD69, so they are less likely to have actively migrated out of the tissue. The possibility that the CD69^−^ population contains ex-TRLs that migrated actively into the perfusate cannot be ruled out.

It is important to consider whether the washout of TRLs is a beneficial or unwanted effect of machine perfusion, given their vital role in local antimicrobial immunity.^15^ Removal of donor leukocytes may reduce organ immunogenicity and reduce posttransplant rejection risk.^8^ This is based on the understanding that, shortly after transplantation, donor antigen-presenting cells can initiate an alloreactive response through direct antigen presentation to recipient immune cells.^28^ Moreover, research has demonstrated that donor-derived CD4^+^ T cells, detected in perfusates and in the peripheral blood of transplant recipients, can activate recipient’s allospecific B cells, leading to donor-specific antibody production.^29,30^ Thus, washing out donor TRLs during machine perfusion might reduce the risk of alloreactive humoral responses. Conversely, removing donor TRLs may weaken local antimicrobial defenses, possibly increasing posttransplant infection risk.^15^ However, given their low proportion in perfusates compared with tissue, the impact of TRL release remains uncertain.^14^

Our study had limitations, including the observational nature and the low number of NMP samples analyzed, especially regarding the correlation analysis and the exclusive inclusion of suboptimal donor kidneys (ie, DCD and DBD-expanded criteria donor, >50 y of age). A larger sample cohort might reveal correlations between the TRL quantities and various clinical characteristics or injury markers. Only 1 case of PNF and AR was observed, limiting the ability to generalize these findings. Additionally, the lack of recorded total perfusate volumes for HMP prevented cell release quantification of HMP samples. The potential impact of up to 48 h of storage time before sample processing on cell viability and surface marker expression is a notable limitation, particularly because of the lack of documentation of exact storage times per sample. Moreover, our study’s reliance on basic phenotypic markers for TRL identification restricted the depth of their phenotypic characterization. Our study was also unable to investigate CD49a ligand release because of the lack of commercially available assays for soluble collagen type I and type IV. Hence, we could not determine whether the release mechanisms for CD49a^+^ TRLs mirrored those of CD103^+^ TRLs.

In conclusion, this observational study demonstrates that during both HMP and NMP of donor kidneys, a diverse repertoire of TRLs is released in perfusates. The release of these cells does not appear to be directly linked to tissue injury but may involve a concurrent loss of their retention ligand.

## Supplementary Material


