# Supplementary material for: Tissue-resident Lymphocytes Are Released During Hypothermic and Normothermic Machine Perfusion of Human Donor Kidneys
Source: Transplantation. 2024 Apr 1;108(7):1551–7. doi: 10.1097/TP.0000000000004936 (PMC11188625; doi:10.1097/TP.0000000000004936)
Supplement: Supplementary file 1 [file tpa-108-1551-s001.pdf]

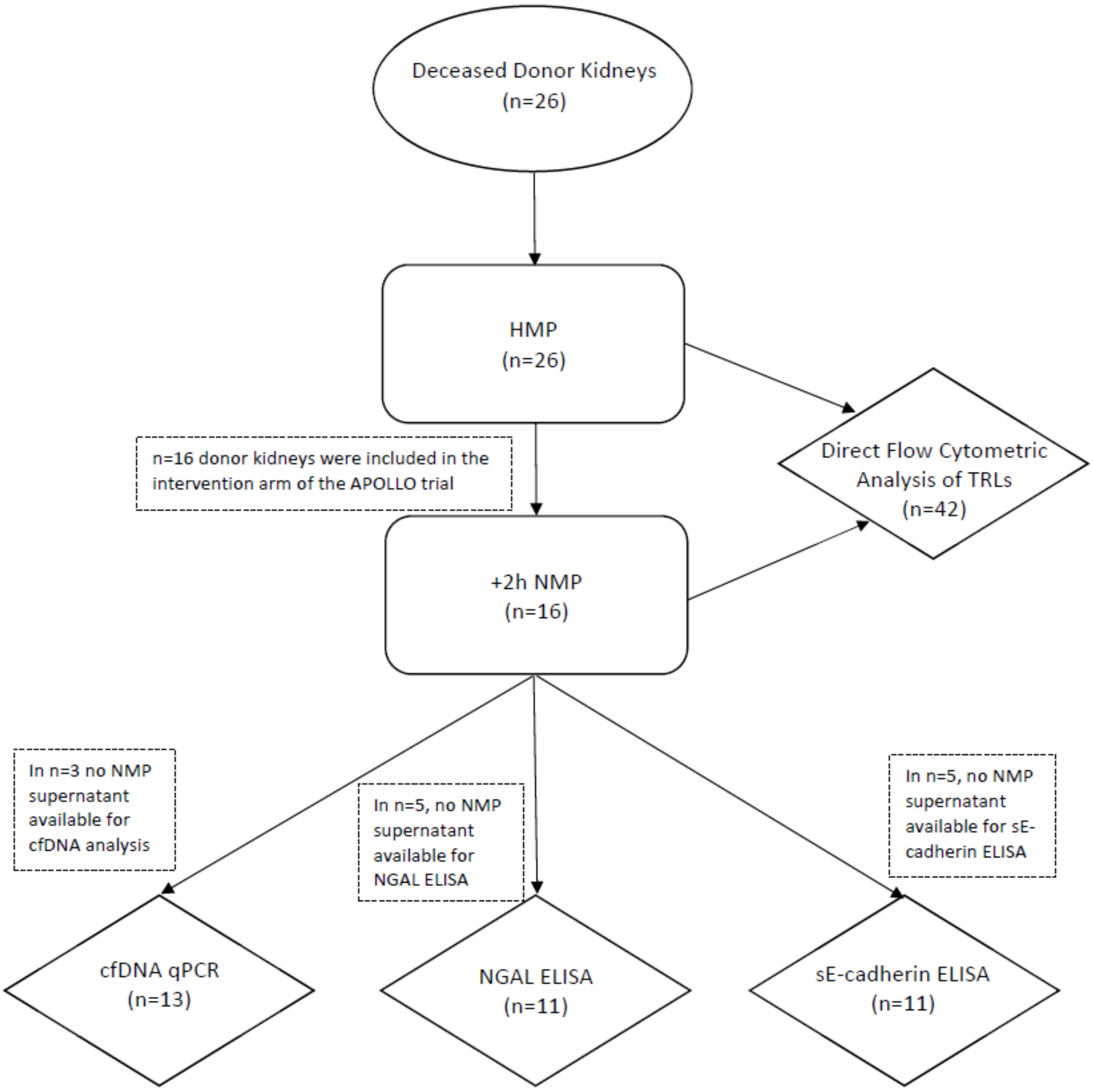

Supplemental Figure S1

Flow chart of included samples and their use in subsequent experiments. HMP = hypothermic machine perfusion. *NMP = normothermic machine perfusion, TRLs = tissue-resident lymphocytes, cfDNA = cell-free DNA, qPCR = quantitative polymerase chain reaction, NGAL = neutrophil gelatinase-associated lipocalin, sE-cadherin = soluble E-cadherin, ELISA = enzyme-linked immunosorbent assay.*

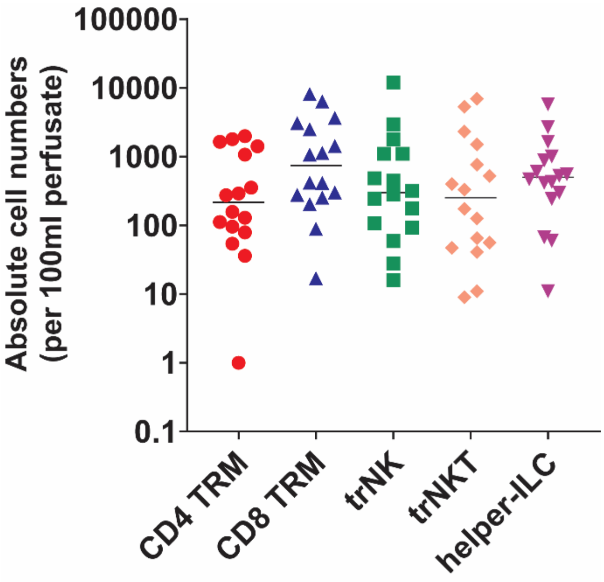

Supplemental Figure S2: Absolute TRL cell counts in NMP perfusates

Quantity of TRL subpopulations per 100ml of NMP samples (n=16). Horizontal bars represent the median. No statistic differences were observed between the groups (as examined using the Mann Whitney U test).

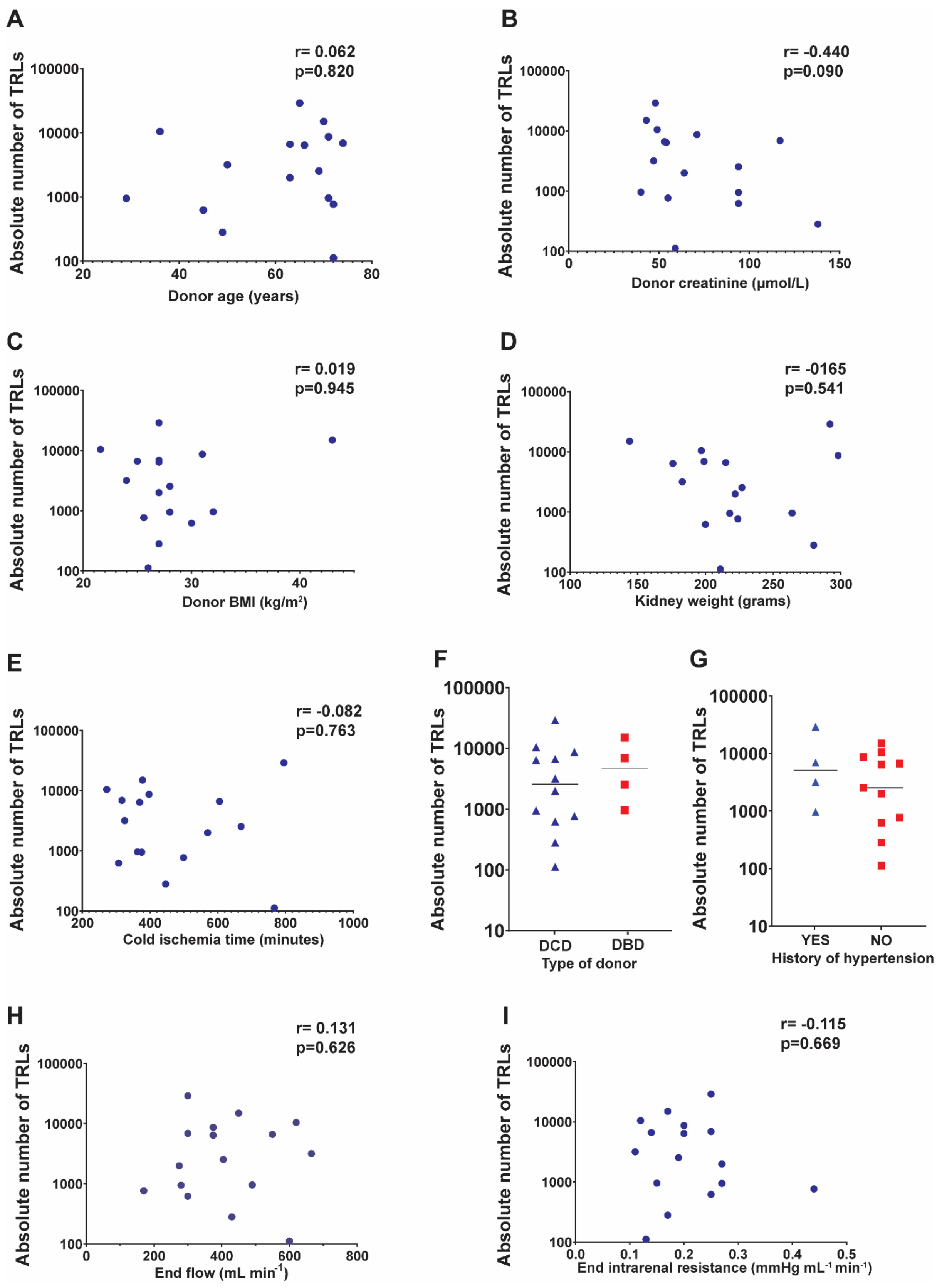
A
r= 0.062
p=0.820
Absolute number of TRLs
Donor age (years)
B
r= -0.440
p=0.090
Absolute number of TRLs
Donor creatinine (µmol/L)
C
r= 0.019
p=0.945
Absolute number of TRLs
Donor BMI (kg/m²)
D
r= -0165
p=0.541
Absolute number of TRLs
Kidney weight (grams)
E
r= -0.082
p=0.763
Absolute number of TRLs
Cold ischemia time (minutes)
F
Absolute number of TRLs
DCD
DBD
Type of donor
G
Absolute number of TRLs
YES
NO
History of hypertension
H
r= 0.131
p=0.626
Absolute number of TRLs
End flow (mL min⁻¹)
I
r= -0.115
p=0.669
Absolute number of TRLs
End intrarenal resistance (mmHg mL⁻¹ min⁻¹)

Supplemental Figure S3 No associations between TRLs in Perfusates and Donor Characteristics or Perfusion Parameters

Correlation analysis between donor characteristics, perfusion parameters and the quantity of TRLs in NMP perfusates (n=16). The quantity of TRLs in NMP samples was not correlated with donor age in years **(A)**, donor creatinine levels **(B)**, donor body mass index (BMI) **(C)**, kidney weight in grams **(D)**, cold ischemia time in minutes **(E)**, type of donor **(F)**, and a history of hypertension **(G)**. The end flow **(H)** and intrarenal resistance **(I)** at the end of perfusion were also not correlated with the quantity of TRLs in NMP samples. Spearman's correlation was used for correlation analysis **(A-E, H, I).** r = correlation coefficient. The Mann-Whitney U test was used to compare two groups **(F-G)**. Horizontal bars represent the median. *P<0.05, **P <0.01, ***P<0.001

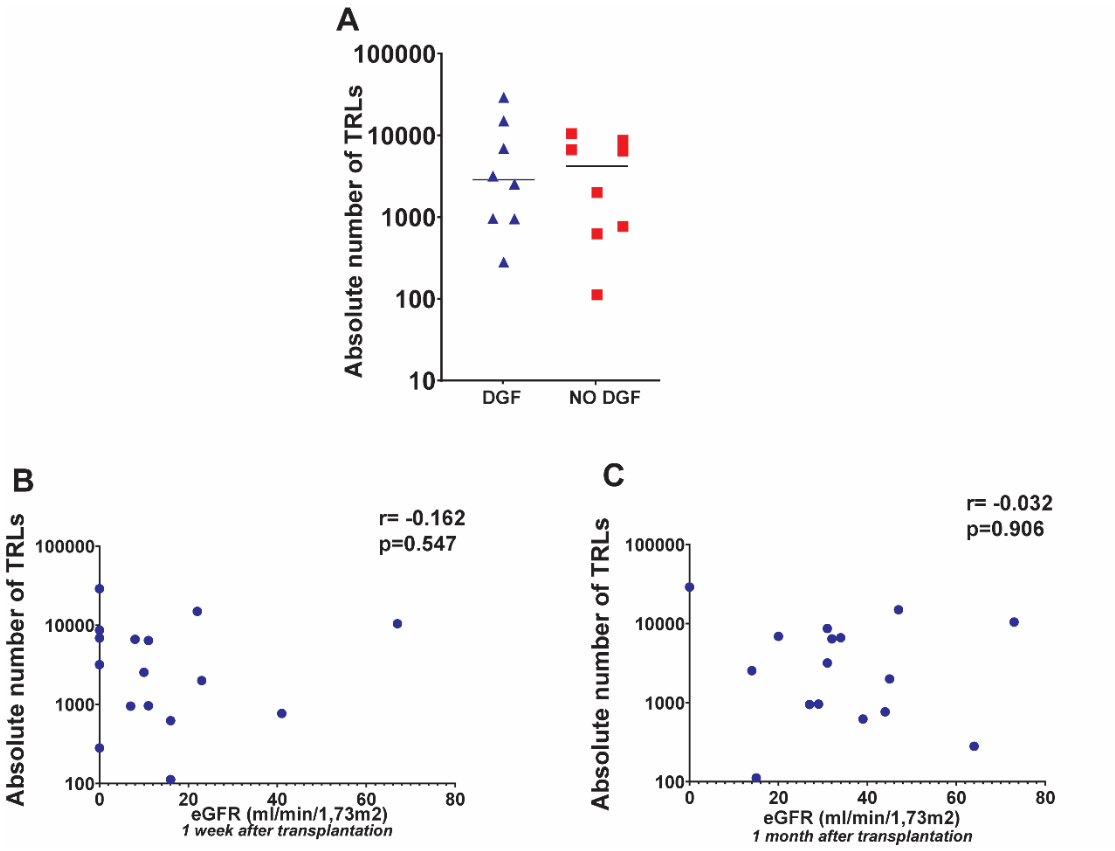

Supplemental Figure S4 No association between TRLs in perfusates and Post-transplant Outcomes

The effect of the quantity of TRL in NMP perfusates (n=16) on post-transplant outcomes was analyzed. There was no difference in the quantity of TRLs in NMP samples and kidneys that developed delayed graft function (DGF) or not **(A)**. No correlation was observed between the amount of TRLs and the eGFR at 1 week **(B)** or 1 month **(C)** post-transplantation. The Mann-Whitney U test was used to compare two groups **(A).** Bars represent the median. Spearman's correlation was used for correlation analysis **(B-C)**. r = correlation coefficient. **P*<0.05, ***P* <0.01, ****P*<0.001

Table S1: Fluorescent Antibodies for Flow Cytometry

Target	Clone	Fluorochrome	Company*
CD4	RPA-T4	PerCP-Cy5	BD Biosciences
CD69	FN50	APC	Biolegend
CD3	UCHT1	AF700	Biolegend
CD45	2D1	BV510	Biolegend
CD8	RPA-T8	BV570	Biolegend
CD11b	ICRF44	BV605	Biolegend
CD16	3G8	BV650	Biolegend
CD14	63D3	BV711	Biolegend
CD127	A019D5	BV785	Biolegend
CD49a	TS2/7	PE	Biolegend
CD56	HCD56	PE/Dazzle	Biolegend
CD19	HIB19	PE-Cy5	Biolegend
CD103	Ber-ACT8	PE-Cy7	Biolegend
Live/dead (Fixable-viability 780)	-	APC-Cy7	BD Biosciences

**BD Biosciences (Franklin Lakes, NJ, USA); Biolegend (San Diego, CA, USA)*

Table S2: Baseline Variables of Donor Kidneys

Variables	HMP (n=26)	NMP (n=16)	p-value
Donor age (years), median (IQR)	63.5 (51.8 – 71.0)	65.5 (49.3 – 71.0)	0.753
Type of donor, (DCD), n (%)	19 (73.1%)	12 (75%)	>0.999
Cold ischemia time (minutes), median (IQR)	529.5 (377.3 – 674.3)	387.5 (334.5 – 596.3)	0.195

*******IQR = interquartile range, DCD = donation after cardiac death, HMP = hypothermic machine perfusion, NMP = normothermic machine perfusion*
